# The Effect of Special Medical Examination for Night Shift Workers and Follow-Up Management Against Hypertension

**DOI:** 10.3390/ijerph16050719

**Published:** 2019-02-28

**Authors:** Won Seon Choi, Ji-Won Lee, Jae Yong Lee, Kyeong Yeon Kim, Jun-Pyo Myong, Won-Chul Lee

**Affiliations:** 1 Department of Occupational and Environmental Medicine, Suwon Center, Korea Medical Institute, Suwon 16571, Korea; cathy302@naver.com; 2 Department of Occupational and Environmental Medicine, Seoul St. Mary’s Hospital, College of Medicine, The Catholic University of Korea, Seoul 06591, Korea; celest2120@gmail.com (J.-W.L.); monsep86@naver.com (J.Y.L.); 3 Department of Occupational and Environmental Medicine, Gwanghwamun Center, Korea Medical Institute, Gwanghwamun 03173, Korea; dr0152@naver.com; 4 Department of Preventive Medicine, Seoul St. Mary’s Hospital, College of Medicine, The Catholic University of Korea, Seoul 06591, Korea

**Keywords:** night shift work, hypertension, follow-up management

## Abstract

Background: Special health examination is a screening program introduced in 1973 in Korea to examine health problems of workers who are regularly exposed to 177 hazardous substances and physical environments specified by the Occupational Safety and Health Act. Shiftwork was added as a risk factor in 2013. The purpose of this study was to analyze changes of hypertension status after a special medical examination and subsequent follow-up management. Methods: We used the data based on the special medical examination outcomes for night shift workers, performed at seven different health examination centers under the Korea Medical Institute (KMI) between 2014 and 2016. Workers who received special medical examinations for two consecutive years (2014–2015 and 2015–2016) were selected. A final study population of 2070 was evaluated. Results: Compared with the first-year examination, 1503 subjects (72.6%) received hypertension medication or showed improvement in blood pressure in their second-year examination. Older age (≥40s), women, larger workplaces (≥300 full-time workers), long-term workers (≥12 years), improvement in smoking habits, improvements for diabetes or dyslipidemia, normal or reduced BMI, and normal waist circumference were associated with proper management of hypertension. Conclusions: An appropriate follow-up management program should be developed to provide health management for night shift workers that need to focus on the factors identified in this study.

## 1. Introduction

In 2017, hypertension was reported to be one of the nine key causes of death in Korea. Hypertension-related diseases account for 5775 deaths annually [1], and hypertension is the key cause of mortalities from cardiovascular and cerebrovascular diseases. Although the cause of essential hypertension, which accounts for most hypertension cases, is unclear, multiple complex factors (e.g., high sodium intake, obesity from high levels of dietary intake, drinking, smoking, lack of physical activities, stress, and age) are speculated to induce hypertension [2]. Aside from factors associated with daily life, shift work is associated with hypertension [3]. Shift work is characterized by non-standardized work (i.e., working outside regular hours between 7–8 AM and 5–6 PM), night shifts, early morning shifts, and rotations [4,5]. Shift work related health issues have been reported because the majority of workers in the fields of production or manufacturing, medical services, and security do shift work. More specifically, shift workers have a higher risk of metabolic syndrome and increased blood pressure. It is suggested that disturbed circadian rhythms, changed lifestyle patterns, and sleep disturbance increase susceptibility and risk of developing cardiovascular diseases [3,6,7]. 

There are two different health examination systems in Korea. One is a special health examination for workers who are exposed to specific risk factors at work, and the other is a general health examination for office workers and non-office workers who are not eligible for the special health examination. In 2013, a special medical examination program for night shift workers was implemented for early detection and management of potential health-related issues arising from working the night shift. This examination is available for the following shift workers: Those working either at least 5 h (including the time between 12 and 5 AM) between 10 PM and 6 AM the next day for six months (a minimum of four times per month) or working >60 h on average between 10 PM and 6 AM the next day for six months [8]. The examination was initially performed on 1 January 2014, for workplaces with ≥300 full-time workers and was expanded to workplaces with 50–299 (since 1 January 2015) and <50 full-time workers (since 1 January 2016). If a disease is discovered after the medical examination, appropriate treatment and follow-up management (i.e., transferring to another department or team) should be performed. Follow-up management is particularly important for chronic diseases that cannot be controlled with short-term treatment. However, the general medical examination that has been most commonly performed in Korea has an important issue where the examinees do not thoroughly read the examination outcome document or are unable to fully understand the content, and consequently are not provided with appropriate follow-up management after the examination [9]. Follow-up management has been emphasized in the special health examination, but effectiveness has not been evaluated. Therefore, additional studies are needed to assess the effect of the special medical examination and subsequent management for night shift workers. 

This study aims to evaluate the improvement of hypertension after a special medical examination and follow-up management, as well as associated factors. The subjects of this study include examinees who underwent a general medical examination in the first year, a special medical examination for night shift workers in the second year, and a follow-up examination (assessment of current blood pressure) on the third year from seven different health examination centers under the Korea Medical Institute (KMI). Small to medium sized companies had relatively poorer conditions in terms of manpower, facilities, and limited resources compared with large sized companies. It is necessary to find efficient methods to improve related factors for hypertension management. By using the data from these subjects, the effectiveness of the special medical examination for night shift workers and follow-up management against hypertension, a representative condition generating the additional risk of cardiovascular diseases, along with associated factors have been investigated.

## 2. Materials and Methods 

**Figure 1:**
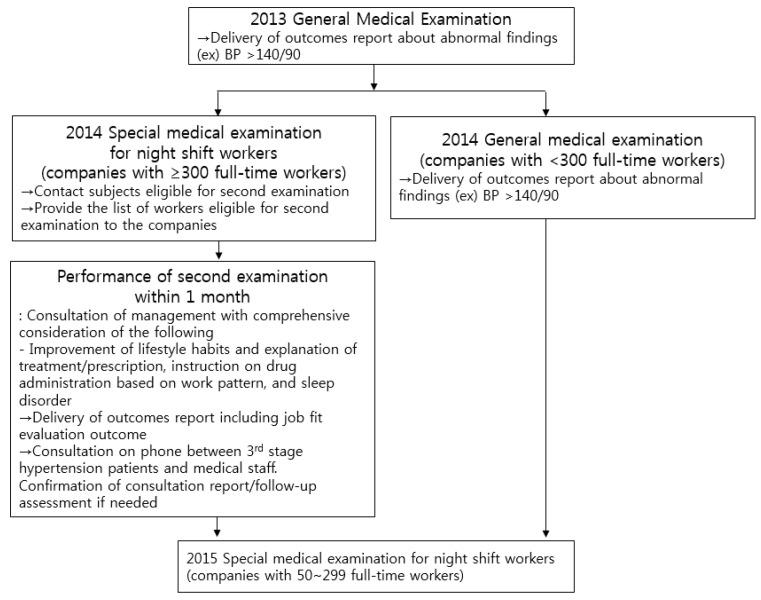
Summary of special medical examination and follow-up process.

The special medical examination for night shift workers is performed annually and includes the assessment of blood pressure, waist circumference, fasting blood sugar, total cholesterol level, triglyceride level, and high-density lipoprotein-cholesterol level, as well as a questionnaire on work history, past medical history, and questions assessing the gastrointestinal, nervous (for sleep disorder), and endocrine systems (for breast diseases). If the worker has a systolic blood pressure (SBP) of ≥140 mmHg or diastolic blood pressure (DBP) of ≥90 mmHg, the worker undergoes a second examination within 1 month at the same medical center. Although each different center might have their own standards, a second examination is performed in cases of severe hypertension (i.e., SBP ≥ 160 mmHg or DBP ≥ 100 mmHg) even if the worker is currently under medication for hypertension. At the second examination, consultation (i.e., for medical or drug treatment) is often provided after the blood pressure measurement with regards to lifestyle habit improvement (i.e., smoking, drinking, dietary habit, and exercise), work patterns, and sleep disorders. If the second visit within 1 month is not feasible due to long travel distance or a new shift work schedule, the worker is instructed to undergo medical treatment at nearby medical centers, and a consultation report is checked. In cases of uncontrollable hypertension, the worker is instructed to be temporarily restricted from additional night shifts. After 2–3 months of intervention, a follow-up assessment is performed to re-evaluate if the worker is fit to work so that the worker (examinee) can manage their blood pressure as much as possible (Figure 1).

In this study, the outcome data of special medical examination for night shift workers, performed at seven health examination centers (Gwanghwamun, Yeouido, Gangnam, Suwon, Gwangju, Daegu, Busan) under KMI between 2014 and 2016, were used for the analysis. The subject’s names and social insurance numbers were removed, and the subjects were given unique identification numbers for the protection of personal information. The study was approved by the Institutional Review Board (IRB) from the Catholic University of Korea (KC17RESI0477).

**Figure 2:**
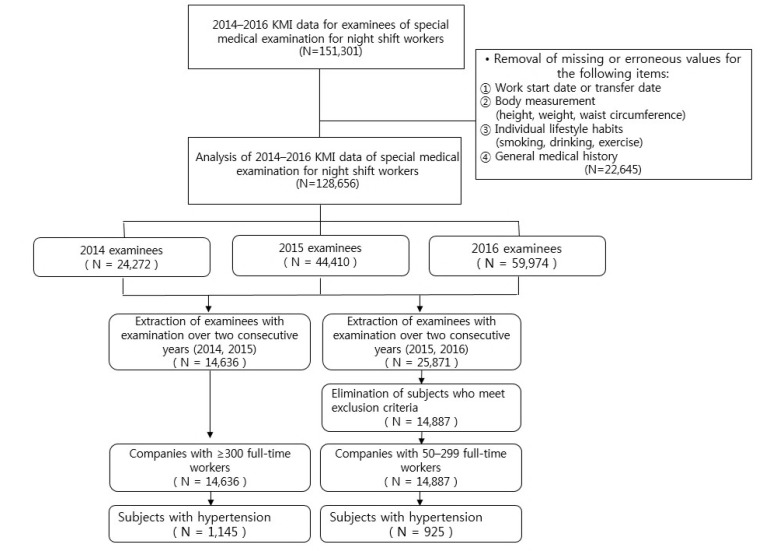
Sampling framework.

From the collected data, the following cases have been removed from the final cohort for analysis: Missing or erroneous information regarding medical history, work start or department transfer date, body measurements (i.e., height, weight, and waist circumference), and personal lifestyle habits (smoking, drinking, and exercise) (N = 151,301). To assess the changes in the management of hypertension based on the special medical examination and subsequent follow-up management, the cases where the worker received special medical examinations for two consecutive years (2014–2015 and 2015–2016) were selected. The sizes of the workplace, based on the Occupational Safety and Health Act, were defined as follows: Workplaces that provided the special medical examination for the first time in 2014 having ≥300 full-time workers (2014–2015, N = 14,636); workplaces that provided the special medical examination for the first time in 2015 having 50–299 full-time workers (2015–2016, N = 14,887) [8]. More specifically, because workers who underwent special medical examinations for two consecutive years (2014–2015 and 2015–2016) had been working night shifts between the two examinations, only subjects who satisfied this condition were considered eligible. Furthermore, to evaluate characteristics of workplaces based on their sizes, the final cohort for analysis excluded the examinees who underwent the special medical examination in 2014 among the examinees who underwent a special medical examination in 2015–2016 (N = 29,523). From this final cohort, the examinees who had underlying hypertension before the special medical examination (N = 2070) were selected for the evaluation of special medical examination and follow-up management outcomes (Figure 2). 

KMI data mainly consisted of demographic characteristics (i.e., sex, age, body measurements (height, weight, and waist circumference), lifestyle habits (drinking, smoking, and exercise), and medical history) and occupational characteristics (i.e., work period and size of companies). 

Based on the body mass index (BMI) calculated using height and weight, the subjects were classified as normal (<23 kg/m^2^), overweight (23–25 kg/m^2^), obese (25–30 kg/m^2^), and severely obese (>30 kg/m^2^). The subjects were divided into two groups based on waist circumference: The abdominal obesity group included male and female subjects with ≥90 cm and ≥85 cm waist circumference, respectively, and the normal group included the remaining subjects. The subjects were also categorized by their smoking habits (current smoker, previous smoker, or non-smoker) and drinking habits (>2 bottles (700 mL) or 14 shots of soju per week for <65-year-old men, >1 bottle(350 mL) or 7 shots of soju per week for ≥65-year-old men and <65-year-old women and half a bottle or 3 shots of soju per week for ≥65-year-old women were considered excessive drinking) [10]. The subjects were determined to have appropriate drinking habits if weekly alcohol consumption was below the standards. Based on the standard of general physical activities from the Korean adult physical activity guideline, the subjects who performed moderate exercise at least three times per week or intense exercise at least twice per week in the last year were categorized into the group with adequate exercise. The remaining subjects were categorized in the group lacking exercise. Work period was calculated in years using the examination date and the current work start date, and the distribution of the entire work period was divided into four quadrants (<2 years, 2–5 years, 5–12 years, and ≥12 years) for further analysis.

Blood pressure measurement and the definition of abnormality in the special medical examination program for night shift workers are as follows: Subjects with SBP ≥ 140 mmHg or DBP ≥ 90 mmHg underwent a second examination for re-assessment of blood pressure. In this study, subjects with SBP ≥ 140 mmHg or DBP ≥ 90 mmHg on their second examination or subjects who should have but did not undergo a second examination (i.e., SBP ≥ 140 mmHg or DBP ≥ 90 mmHg on the first examination) were considered to exhibit hypertension. For the analysis of follow-up management, the following subjects, of those that underwent special medical examination for ≥2 years, were considered to have undergone appropriate follow-up management: The subjects who had hypertension on the first year’s examination but did not on the second year’s examination (i.e., SBP < 140 mmHg and DBP < 90 mmHg) or who responded that they were receiving appropriate hypertension management on the second year’s examination survey. Factors affecting appropriate follow-up management included individual characteristics and lifestyle habits (i.e., exercise, drinking, smoking, comorbidities, BMI, and waist circumference), and the changes in these factors over 1 year were variables used to compare the two groups with and without appropriate follow-up management (Table 1).

To investigate whether appropriate follow-up management is effective for improving the subjects diagnosed with hypertension in the first year, univariate analysis (χ^2^-test) was performed based on individual and occupational characteristics. Using the individual characteristics, comorbidities, and lifestyle habit improvement confirmed in the second-year examination as variables, the odds ratio (OR) and 95% confidence interval (CI) of appropriate follow-up hypertension management were calculated using multiple logistic regression analysis. Furthermore, to assess the adequacy of follow-up hypertension management based on the size of companies, the data were stratified, and multiple logistic regression analyses were performed. Data cleansing/processing and general statistical analyses were performed using SAS 9.4 (SAS Institute, Cary, NC, USA). 

## 3. Results

### 3.1. Follow-Up Management of the Subjects with Hypertension

The subjects diagnosed with hypertension in the first-year examination were considered to be properly managed if the subjects were receiving medication or have reduced blood pressure (below the standard) in the second-year examination. Overall, 1530 of 2070 subjects (72.6%) were managed properly, whereas the remaining 567 subjects (27.4%) were not managed appropriately (Table 2).

In the comparison based on age groups, the younger group (≤30s) had a relatively higher proportion of subjects who did not have appropriate blood pressure management, whereas the older group (≥40s) had a significantly higher proportion of subjects with appropriate blood management. The proportion of women in the proper management group (9.5%) was small but significantly higher compared with the improper management group (6.2%). 

By the size of companies, the proportion of subjects with proper management was significantly higher (57.7%) for larger workplaces (≥300 full-time workers). Furthermore, a greater proportion of long-term workers (≥12 years) had proper management of hypertension (39.6%) compared with short-term workers.

The number of subjects with improvement in smoking habits in the proper management group (988 subjects, 65.7%) was significantly higher than that in the improper management group (60.1%). Similarly, there was a higher number of subjects with improved exercise and drinking habits in the proper management group (55.9%, 65.9%), although the difference was not statistically significant. 

In case of comorbidities, the subjects with improvements for diabetes or dyslipidemia showed a significantly greater tendency of hypertension management. 

The analysis between BMI and management of hypertension showed a greater proportion of subjects with a normal level of BMI or reduced obesity in the proper management group. In the analysis between waist circumference and hypertension, a greater proportion of subjects had maintained normal circumference in the proper management group.

### 3.2. Characteristics of Subjects with Hypertension in the Proper Management Group

Multiple logistics analyses of the subjects under the management of hypertension in the second-year examination showed that increasing age was associated with greater OR for the subjects being properly managed. More specifically, female and long-term workers (≥12 years) had proper management of hypertension (Table 3). 

From the comparison by the size of companies, the companies with 50–299 full-time workers exhibited significantly greater improper management of hypertension with an OR of 0.71 (95% CI, 0.58–0.86), even after adjusting for other factors (OR, 0.68, 95% CI, 0.55–0.83).

With regards to the improvement of individual lifestyle habits, the OR was slightly increased in the group with improvement in all assessed factors (i.e., exercise, drinking, or smoking), although the difference was not statistically significant. The groups with diabetes or dyslipidemia as comorbidities had ORs of 10.74 (95% CI, 3.25–37.32) and 4.55 (95% CI, 2.21–11.03) in the groups with an improvement of the condition, indicating that the groups with comorbidities being managed had more proper management of hypertension. 

Similarly, the subjects with normal weight maintained (OR, 1.48; 95% CI, 1.04–2.12) or reduced obesity compared with the first examination (OR, 1.35; 95% CI, 1.06–1.72) had a greater possibility of hypertension improvement. In addition, the subjects with maintained waist circumference had the greatest OR (OR, 1.61; 95% CI, 1.23–2.12). 

### 3.3. Characteristics of the Subjects in Proper Management Group Based on the Size of Companies

The analysis of stratified data based on the size of companies showed that female and aged workers working at companies with ≥300 full-time workers had a greater tendency of proper hypertension management, although the difference was not statistically significant (Table 4). Meanwhile, no difference was found based on the work period. Although no significant changes were found based on the improvement of lifestyle habits (i.e., drinking, smoking, or exercise), the groups with improvements in diabetes or dyslipidemia had ORs of 3.62 (95% CI, 1.247–15.374) and 13.35 (95% CI, 2.828–238.686), respectively, indicating that these groups exhibited proper management of hypertension. Furthermore, although not statistically significant, the subjects with reduced obesity or maintained normal weight showed more proper management of hypertension. Similarly, only the group of subjects with maintained normal waist circumference showed a significant outcome (OR, 1.51; 95% CI, 1.053–2.158).

In the companies with 50–299 full-time workers, the OR was significantly lower for the workers in their 30s at 0.56 compared with the workers in their 20s (95% CI, 0.329–0.929), and was higher in their 40s and 50s. Even after adjusting for other variables, female subjects showed more proper management of hypertension. Although the OR of proper follow-up management was greater for workers who worked for ≥2 years compared with those who worked for <2 years, the difference was not statistically significant. Furthermore, no significant association was observed between proper management of hypertension and improvement in lifestyle habits (i.e., exercise, drinking, and smoking). For workers with diabetes or dyslipidemia, the group of subjects with improved comorbidities had more proper management of hypertension, similar to the companies with ≥300 full-time workers (diabetes OR, 5.16; 95% CI, 2.024–17.485; dyslipidemia OR, 3.90; 95% CI, 1.092–24.958). The OR for obesity and waist circumference was higher in the group with improvement or maintained, but the difference was not significant after adjusting for other variables.

## 4. Discussion

The aim of this study is to assess the changes after the special medical examination and follow-up management in the group of subjects who underwent general medical examinations before the special medical examination for night shift workers and show matched outcomes from the special medical examination. Among the total of 2070 subjects of the initial cohort, 1503 subjects (72.6%) were receiving medication or showing improved blood pressure in their second-year examination compared with the first-year examination. 

The improvement of the subjects with hypertension (72.6%) after a special medical examination and follow-up management may be due to the following: First, the follow-up management outcome may seem favorable due to the unique characteristics of the special medical examination for night shift workers. Night shift workers undergo an annual special medical examination before and after the initial special medical examination. Whereas the traditional general medical examination outcomes are simply shown in a paper format, both first and second special medical examinations are performed at an identical medical center, and they provide a job fit evaluation and appropriate follow-up management. Second, the special medical examination for night shift workers is performed at the same medical center, within one month from the first special examination. In addition, follow-up management is also performed at the same center, allowing for continuous support, active intervention of medical staff, and cooperation of workers to evaluate job fit. Third, with workers eligible for traditional general medical examinations being newly eligible for special examination, the workers may have become more aware of the risk of cardiovascular diseases from night shift work and thus improved in the second-year examination. According to the Knowledge–Attitude–Practice (KAP) model, accumulation of knowledge brings change in the attitude and eventually leads to favorable health practices. Therefore, the recognition of risk by the workers during the examination process may have caused them to better manage their health condition [11].

Smaller sizes of companies, with less full-time workers, were associated with a lower improvement rate of hypertension after special medical examination. The salary of workers for mid-size companies in Korea is approximately 53.6% of that for big corporations [12], and a larger proportion of workers in mid-size companies are middle-aged or older [13]. The operation of mid-sized companies is often limited by a relative shortage of professional workforce and resources, and thus implementing a well-structured health promotional program is difficult [14,15]. Moreover, the workers are economically and temporally limited to health management [16]. These limitations of mid-size companies may have been the cause for inappropriate health management of the workers. Therefore, additional measures (i.e., follow-up management after the specialized medical examination for night shift workers) for mid-sized companies, with relatively poorer conditions and limited resources compared with big corporations, may be needed.

In this study, not only the presence of comorbidities (diabetes and dyslipidemia) but also an improvement compared with the previous year’s examination was evaluated. The analysis outcomes indicated that the subjects under medical treatment or proper management exhibited improvement in blood pressure condition (diabetes OR, 10.74; 95% CI, 3.25–37.32; dyslipidemia OR, 4.55; 95% CI, 2.21–11.03). Workers with multiple diseases more frequently visited the medical center and had a greater awareness of hypertension, and therefore were more capable of blood pressure management [17,18]. Furthermore, hypertension, diabetes, and dyslipidemia all require improvement of lifestyle habit (i.e., diet, exercise, weight management, etc.), and therefore, the management of these comorbidities may have assisted with the management of hypertension.

All night shift workers (10 PM–6 AM) are eligible for a special medical examination. There are various types of night shift workers: Fixed night shift workers, double-shift workers, and triple-shift workers. Several previous studies have reported that shift workers have an increased risk of obesity, hypertension, dyslipidemia, and diabetes [19,20,21,22]. A cross-sectional study on Korean nurses has demonstrated that more long-term shift workers were overweight or obese compared with short-term shift workers [20,23]. In this study, the management of hypertension was not different based on the work period. This is likely due to the nature of jobs that require a night shift in Korea (i.e., securities or facility management jobs) being short-term contracts or outsourced, and thus the result of the survey may not accurately represent the actual work period. Future studies should assess and consider possible early job or position changes in the workers.

The OR for proper management of blood pressure was higher in the group with improvement in obesity or normally maintained weight (Table 2). Being overweight or obese is a well-known risk factor for hypertension, and a retrospective study that analyzed blood pressure measurements over a 6-month period in 395 subjects showed that overweight or obese groups demonstrated poorer control of blood pressure compared with normal or underweight groups [24]. In addition, studies from Spain and China have also shown that overweight or obese subjects exhibit poorer management of hypertension [25,26]. Therefore, weight management should be the focus of follow-up management after a special medical examination for night shift workers. 

In this study, the subjects in their 40s and 50s showed a tendency for better management of hypertension (Table 2). A previous study analyzing 1999–2004 U.S. National Health and Nutrition Survey has mentioned that the subjects in their 60s or older had greater rates of recognition, treatment, and management for hypertension [27]. Another cross-sectional study on 2445 patients from Hong Kong has demonstrated that older subjects have increased drug compliance [28], which was likely because older subjects use medical services and measure blood pressure more often with an increased interest in their own health. In contrast, subjects with hypertension in their 30s and 40s from another Korean study that analyzed the prevalence of hypertension and treatment status exhibited very low rates of management (8% for 30s and 26% for 40s) [29]. These subjects rarely use medical centers, have wrongful information that they will need to be on medication for the rest of their lives, and exhibit poor management because they do not feel the need for treatment of an asymptomatic condition. Therefore, focusing on older subjects may be a more efficient strategy for follow-up management when performing multiple follow-up managements within a limited timeframe. Furthermore, specialized consultation strategy for younger subjects should also be performed.

There are several limitations in the present study. Since the conclusions were deduced based on the self-administrated survey of lifestyle habit improvement, the factor associated with improvement could not be directly confirmed. In addition, because the comparison was made in one year, there are limitations in determining the effect of blood pressure improvement over such a relatively short period. The difference according to different types of work and shift work pattern could not be assessed due to lack of information on occupational characteristics, and small companies (<50 full-time workers) started the examination in 2016 and thus were not included in the study. Future studies should evaluate these smaller companies. Sleep disturbance was not included in the analysis due to the low reliability of the survey. Overall, a well-structured follow-up management program should be developed using the factors identified in this study to provide health management for night shift workers who work under special conditions. In spites of those limitations, the present study has several strengths such as relatively large sample size and control for potential bias with stratification based on BMI and waist circumference.

## 5. Conclusions

This study has confirmed the effectiveness of the special medical examination for night shift workers in the companies with 50 or more employees (since 2014) for the management of hypertension in multi-center for medical examination. 

## Figures and Tables

**Table 1:** Factors associated with follow-up management.

	Maintained	Improved	Not Improved
Drinking	-	Adequate level on the second-year survey	Excessive drinking on the second-year survey
Exercise	-	Adequate level on the second-year survey	Lacking exercise on the second-year survey
Smoking	-	Non-smoker or previous smoker on the second-year survey	Current smoker on the second-year survey
Comorbidities			
Diabetes	Not diagnosed with diabetes on either first- or second-year examination	Second examination outcomes in the first year showing fasting blood sugar level of ≥126 mg/dL or HbA1c level of ≥6.5%, but blood test in the second-year examination showing fasting blood sugar level of <126 mg/dL or HbA1c level of <6.5% or the patient was currently under anti-diabetic medications	Second examination outcomes in both first and second years showing fasting blood sugar level of ≥126 mg/dL or HbA1c level of ≥6.5%
Dyslipidemia	Not diagnosed with dyslipidemia on either first- or second-year examination (blood test)	Second examination outcomes (blood test) in the first year showing LDL cholesterol level of ≥160 mg/dL or triglyceride level of ≥500 mg/dL or total cholesterol level of ≥240 mg/dL, but blood test in the second year showing the levels below the standard of dyslipidemia or the patient currently under treatment improved	Second examination outcomes on both first- and second-years showing LDL cholesterol level of ≥160 mg/dL or triglyceride level of ≥500 mg/dL or total cholesterol level of ≥240 mg/dL
BMI *	Normal BMI on both first and second years (<23)	BMI in the second year decreased compared with BMI in the first-year examination	BMI in the second year increased compared with BMI in the first-year examination
Waist circumference *	Normal waist circumference on both first- and second-year examinations (<90 cm for men and <85 cm for women)	Waist circumference measured in the second year decreased compared with waist circumference measured in the first-year examination	Waist circumference measured in the second-year examination increased compared with waist circumference measured in the first-year examination

* “Maintained” if the values measured in the first-year examination were the same as those measured in the second-year examination.

**Table 2:** Characteristics of subjects and management of hypertension.

	Improper, N (%) (n = 567 (27.4%))	Proper, N (%) (n = 1503 (72.6%))	*p*-Value *
Age *			<0.0001
≤20 s	63 (11.1)	125 (8.3)	
30 s	198 (34.9)	360 (24.0)	
40 s	174 (30.7)	507 (33.7)	
≥50 s	132 (23.3)	511 (34.0)	
Sex *			0.0156
Male	532 (93.8)	1360 (90.5)	
Female	35 (6.2)	143 (9.5)	
Size of companies ^†^			0.0004
≥300	278 (49.0)	867 (57.7)	
50–299	289 (51.0)	636 (42.3)	
Work period			0.0093
<2 years	68 (12.1)	129 (8.7)	
2–5 years	167 (29.7)	420 (28.3)	
5–12 years	144 (25.6)	348 (23.4)	
≥12 years	183 (32.6)	589 (39.6)	
Exercising			0.4766
Improved	307 (54.1)	840 (55.9)	
Not improved	260 (45.9)	663 (44.1)	
Drinking habit			0.5237
Improved	365 (64.4)	990 (65.9)	
Not improved	202 (35.6)	513 (34.1)	
Smoking habit *			0.0179
Improved	341 (60.1)	988 (65.7)	
Not improved	226 (39.9)	515 (34.3)	
Diabetes			<0.0001
Maintained	552 (97.4)	1399 (93.1)	
Improved	7 (1.2)	94 (6.3)	
Not improved	8 (1.4)	10 (0.7)	
Dyslipidemia			<0.0001
Maintained	561 (98.9)	1428 (95.0)	
Improved	3 (0.5)	67 (4.5)	
Not improved	3 (0.5)	8 (0.5)	
BMI *			<0.0001
Normal	58 (10.2)	252 (16.8)	
Improved	186 (32.8)	563 (37.5)	
Maintained	48 (8.5)	102 (6.8)	
Not improved	275 (48.5)	586 (39.0)	
Waist circumference			<0.0001
Normal	252 (44.4)	870 (57.9)	
Improved	125 (22.1)	280 (18.6)	
Maintained	37 (6.5)	64(4.3)	
Not improved	153 (27.0)	289(19.2)	

* by χ^2^-test. ^†^ Number of employed full-time workers.

**Table 3:** Association between general and work-related factors and proper management of hypertension.

Variables		Before Adjustment		* After Adjustment	
		OR	95% CI	OR	95% CI
Age					
	≤20 s	1.00		1.00	
	30 s	0.92	0.64–1.30	0.82	0.56–1.19
	40 s	1.47	1.03–2.08	1.13	0.76–1.67
	≥50 s	1.95	1.36–2.79	1.44	0.96–2.15
Sex					
	Male	1.00		1.00	
	Female	1.60	1.10–2.38	1.34	0.90–2.05
Work period					
	2 years	1.00		1.00	
	5 years	1.33	0.94–1.87	1.30	0.90–1.86
	12 years	1.27	0.89–1.81	1.25	0.85–1.81
	12 years	1.70	1.21–2.37	1.36	0.93–1.96
Size of companies ^†^					
	≥300	1.00		1.00	
	50–299	0.71	0.58–0.86	0.68	0.55–0.83
Exercising					
	Not improved	1.00		1.00	
	Improved	1.07	0.88–1.30	1.02	0.83–1.25
Drinking					
	Not improved	1.00		1.00	
	Improved	1.07	0.87–1.31	0.97	0.78–1.21
Smoking					
	Not improved	1.00		1.00	
	Improved	1.27	1.04–1.55	1.04	0.84–1.30
Diabetes					
	Not improved	1.00		1.00	
	Improved	10.74	3.25–37.32	4.55	2.21–11.03
	Maintained	2.03	0.77–5.17	0.52	0.19–1.43
Dyslipidemia					
	Not improved	1.00		1.00	
	Improved	8.37	1.36–52.65	6.97	2.54–28.86
	Maintained	0.96	0.21–3.31	0.98	0.26–4.69
BMI					
	Not improved	1.00		1.00	
	Maintained	1.00	0.69–1.46	0.89	0.60–1.32
	Improved	1.42	1.14–1.77	1.35	1.06–1.72
	Maintained normal	2.04	1.49–2.83	1.48	1.04–2.12
Waist circumference					
	Not improved	1.00		1.00	
	Maintained	0.92	0.59–1.45	0.84	0.52–1.34
	Improved	1.19	0.89–1.58	1.10	0.80–1.52
	Maintained normal	1.83	1.44–2.32	1.61	1.23–2.12

^†^ Number of employed full-time workers; OR: odd ratio, CI: confidential interval. * Adjusted for age, sex, work duration, improved status of alcohol drinking, smoking, physical activity, diabetes mellitus, dyslipidemia, body mass index, and waist circumference.

**Table 4:** Comparison of proper management of hypertension by size of companies (N = 2070).

	≥300 (N = 1145)		50–299 (N = 925)	
	Unadjusted	Adjusted *	Unadjusted	Adjusted *
Age				
≤20 s	1.00	1.00	1.00	1.00
30 s	1.16 (0.70–1.89)	1.22 (0.73–2.00)	0.63 (0.38–1.04)	0.56 (0.33–0.93)
40 s	1.39 (0.84–2.25)	1.33 (0.80–2.19)	1.47 (0.88–2.42)	1.24 (0.73–2.09)
≥50 s	1.55 (0.92–2.59)	1.46 (0.85–2.50)	2.45 (1.48–4.02)	2.04 (1.19–3.47)
Sex				
Males	1.00	1.00	1.00	1.00
Females	1.18 (0.72–1.99)	1.11 (0.67–1.94)	2.31 (1.32–4.35)	1.87 (1.02–3.65)
Work period				
<2 years	1	1	1	1
2–5 years	1.54 (0.85–2.73)	1.58 (0.85–2.86)	1.09 (0.70–1.67)	1.21 (0.76–1.92)
5–12 years	1.03 (0.57–1.81)	1.01 (0.54–1.83)	1.45 (0.91–2.32)	1.65 (0.99–2.76)
≥12 years	1.58 (0.89–2.73)	1.43 (0.77–2.57)	1.54 (0.99–2.41)	1.37 (0.84–2.24)
Exercising				
Not improved	1.00	1.00	1.00	1.00
Improved	1.06 (0.81–1.39)	1.02 (0.77–1.35)	1.09 (0.82–1.44)	0.97 (0.72–1.31)
Drinking				
Not improved	1.00	1.00	1.00	1.00
Improved	0.90 (0.68–1.19)	0.81 (0.61–1.08)	1.40 (1.04–1.89)	1.15 (0.84–1.58)
Smoking				
Not improved	1.00	1.00	1.00	1.00
Improved	1.22 (0.92–1.61)	1.17 (0.87–1.58)	1.29 (0.97–1.71)	0.90 (0.65–1.24)
Diabetes				
Not improved	1.00	1.00	1.00	1.00
Improved	<0.001(7.85))	3.62(1.25–15.37)	22.80(5.41–116.02)	5.16 (2.02–17.49)
Maintained normal	<0.001(1.41)	>999.999(0.48)	3.32(1.10–11.06)	0.35 (0.10–1.14)
Dyslipidemia				
Not improved	1.00	1.00	1.00	1.00
Improved	6.67 (0.24–184.72)	13.35(2.83–238.69)	13.50 (1.16–189.52)	3.90 (1.09–24.96)
Maintained normal	0.50 (0.03–2.92)	1.94 (0.31–37.42)	2.13 (0.25–17.82)	0.62 (0.07–5.70)
BMI				
Not improved	1.00	1.00	1.00	1.00
Maintained	0.93 (0.56–1.58)	0.85 (0.50–1.47)	1.09 (0.64–1.88)	0.94 (0.54–1.68)
Improved	1.35 (1.00–1.83)	1.37 (0.99–1.91)	1.48 (1.08–2.04)	1.33 (0.93–1.91)
Maintained normal	2.07 (1.32–3.35)	1.57 (0.96–2.64)	2.06 (1.34–3.25)	1.48 (0.91–2.46)
Waist circumference				
Not improved	1.00	1.00	1.00	1.00
Maintained	0.85 (0.47–1.60)	0.85 (0.46–1.61)	1.02 (0.52–2.01)	0.80 (0.39–1.63)
Improved	0.95 (0.64–1.43)	0.85 (0.55–1.31)	1.61 (1.06–2.44)	1.48 (0.93–2.37)
Maintained normal	1.73 (1.24–2.40)	1.51 (1.05–2.16)	2.05 (1.43–2.92)	1.41 (0.93–2.11)

Data are shown as odds ratios (95% confidence interval). * Adjusted for age, sex, work duration, improved status of alcohol drinking, smoking, physical activity, diabetes mellitus, dyslipidemia, body mass index, and waist circumference.
